# In Vitro Killing of Canine Urinary Tract Infection Pathogens by Ampicillin, Cephalexin, Marbofloxacin, Pradofloxacin, and Trimethoprim/Sulfamethoxazole

**DOI:** 10.3390/microorganisms9112279

**Published:** 2021-11-02

**Authors:** Joseph M. Blondeau, Shantelle D. Fitch

**Affiliations:** 1 Departments of Microbiology and Immunology, Pathology and Laboratory Medicine and Ophthalmology, University of Saskatchewan, Saskatoon, SK S7N 0W8, Canada; 2 Department of Clinical Microbiology, Royal University Hospital and Saskatchewan Health Authority, Saskatoon, SK S7N 0W8, Canada; shantelle.fitch@saskhealthauthority.ca

**Keywords:** urinary tract pathogens, antibiotics, in vitro killing

## Abstract

Urinary tract infections are common in dogs, necessitating antimicrobial therapy. We determined the speed and extent of in vitro killing of canine urinary tract infection pathogens by five antimicrobial agents (ampicillin, cephalexin, marbofloxacin, pradofloxacin, and trimethoprim/sulfamethoxazole) following the first 3 h of drug exposure. Minimum inhibitory and mutant prevention drug concentrations were determined for each strain. In vitro killing was determined by exposing bacteria to clinically relevant drug concentrations and recording the log_10_ reduction and percent kill in viable cells at timed intervals. Marbofloxacin and pradofloxacin killed more bacterial cells, and faster than other agents, depending on the time of sampling and drug concentration. Significant differences were seen between drugs for killing *Escherichia coli*, *Proteus mirabilis*, *Enterococcus faecalis*, and *Staphylococcus pseudintermedius* strains. At the maximum urine drug concentrations, significantly more *E. coli* cells were killed by marbofloxacin than by ampicillin (*p* < 0.0001), cephalexin (*p* < 0.0001), and TMP/SMX (*p* < 0.0001) and by pradofloxacin than by cephalexin (*p* < 0.0001) and TMP/SMX (*p* < 0.0001), following 5 min of drug exposure. Rapid killing of bacteria should inform thinking on drug selection for short course therapy for uncomplicated UTIs, without compromising patient care, and is consistent with appropriate antimicrobial use and stewardship principles.

## 1. Introduction

Urinary tract infections (UTI) or sporadic bacterial cystitis (SBC) are common in dogs and less frequently seen in cats [1]. Up to 14% of all dogs may experience a UTI during their lifetimes; this increases in frequency with older age [2] or following some surgeries [3]. In cats, the frequency of UTIs is less than 1% and increases in frequency with advancing age. In many animals, UTIs may be undiagnosed and discovered as an incidental finding; however, the consequences of untreated infections include lower urinary tract dysfunction, urolithiasis, prostatitis, infertility, septicaemia, and pyelonephritis [4,5], although some of these complications are rare. Findings associated with uncomplicated infections may include pollakiuria, dysuria, urgency, straining, and urinary accidents.

The pathogenesis of uncomplicated cystitis is complex and is primarily seen in female patients due to the relatively short urethra [6]. *Escherichia coli* (*E. coli*) remains the single most common pathogen in acute and recurrent UTI; however, pathogens, such as *Staphylococcus pseudintermedius*, *Proteus* species (spp.), and other Gram-negative bacilli, *Streptococcus* spp., and *Enterococcus* spp., may cause infection. Collectively, *E. coli*, *Staphylococcus* spp., *Proteus* spp., and *Enterococcus* spp. account for more than 70% of bacterial causes of UTI in dogs [7].

Antimicrobial therapy remains the cornerstone for UTI treatment. Treatment guidelines for SBC (dogs and cats) recommend beta-lactams (including cephalosporins), fluoroquinolones, doxycycline, and trimethoprim/sulfadiazine or sulfamethoxazole with therapy duration of 7 days or longer [8]; however, the more recently published guidelines, in fact, recommend 3–5 days of therapy [1]. Currently amoxicillin and TMP/SMX are recommended as first line agents for empiric therapy; however, randomized controlled trials supporting these recommendations are limited. The authors acknowledge shorter durations of therapy are likely possible, but relevant randomized control trial and in vitro data is minimal. At least two studies in dogs showed short course therapy was not inferior to longer treatment durations. Clare et al. (2014) reported 3 days of therapy with TMP/SMX (15 mg/kg q12 orally) was equivalent to 10 days of cephalexin (20 mg/kg q12 orally) in 38 female dogs with UTI [9]. It was shown that 3 days of enrofloxacin (18–20 mg/kg q24 orally) therapy was not inferior to 14 days of amoxicillin/clavulanic acid (13.75–25 mg/kg q12 orally) in otherwise healthy dogs, with clinical evidence of cystitis [10]. Shorter durations of therapy for uncomplicated UTIs in humans have been standard for many years without compromising patient care. For example, a summary report indicated that 5 days of therapy with nitrofurantoin for acute uncomplicated cystitis was as effective as is 3 days of therapy with fluoroquinolones (i.e., ciprofloxacin, levofloxacin) or trimethoprim/sulfamethoxazole (TMP/SMX) [11]. By comparison, beta-lactam agents (amoxicillin–clavulanate, cefdinir, cefaclor, and others) for 3–7 days are appropriate choices, but beta-lactam agents have inferior efficacy in general, and more adverse events when compared to other antimicrobials used to treat UTIs in humans [12,13,14,15]. The efficacy of 3-day ciprofloxacin therapy in elderly women was not inferior to 7 days of ciprofloxacin and was better tolerated [16].

In vitro investigations based on the minimum inhibitory concentration (MIC) have (and continue to be) used to compare and contrast antimicrobial agents for antimicrobial potency [17]. The propensity of an antimicrobial agent to select for resistance can be based on the mutant prevention concentration (MPC) [17,18,19]—an in vitro measurement based on testing higher inocula of bacteria to determine the drug concentration blocking growth of the least susceptible cells in high-density bacterial populations. The speed and extent of killing or inhibition of growth are based on time-kill studies [20,21,22]. Drugs are categorized as either bactericidal (kill) or bacteriostatic (inhibition) based on previously published criteria or definitions; however, the clinical importance of such designation continues to be debated [20,23,24,25] and may be irrelevant.

In this report, we measured MIC and MPC values for key companion animal wild type urinary tract pathogens and used four clinically relevant drug concentrations, including the maximum serum (relevant for sepsis) and urine drug concentrations in 3-h time kill assays to determine the speed and extent of killing by five antimicrobial agents shortly after drug exposure. Three of the tested agents could be considered for short course UTI therapy if supported by clinical trial data.

## 2. Materials and Methods

### 2.1. Bacterial Strains

Three non-duplicate clinical isolates, each of *E. coli*, *S. pseudintermedius*, *P. mirabilis*, and *E. faecalis*, collected from canine urine specimens at Prairie Diagnostic Laboratory, Western College of Veterinary Medicine, University of Saskatchewan, Saskatoon, SK, Canada were used. Organism identification was by VITEK II (BioMerieux, St. Laurent, QC, Canada) and confirmed by matrix-assisted laser desorption ionization—time of flight (MALDI-TOF) (BioMerieux, St. Laurent, QC, Canada). Isolates were cultured on tryptic soy agar containing 5% sheep red blood cells (BA) (Oxoid, Nepean, ON, Canada) in O_2_ at 35–37 °C for 18–24 h. Single colonies were selected and transferred to skim milk and stored frozen at −70 °C. No pre-selection criteria favoured the inclusion of organisms with specific susceptibility to any drug tested; however, each isolate had to be susceptible to each agent based on available recommended susceptibility MIC breakpoints [26].

### 2.2. Antimicrobial Compounds

Pure substance pradofloxacin was obtained from Bayer Animal Health (Elanco, as of 2020) and prepared as per the manufacturer’s instructions. Ampicillin (Auro Pharma, Woodbridge, ON, Canada), cephalexin (Novapharm, Scarborough, ON, Canada), marbofloxacin (Zoetis, Kirkland, QC, Canada), and trimethoprim/sulfamethoxazole (TMP/SMX) (Sigma Aldrich, Oakville, ON, Canada) were purchased commercially and prepared in accordance with the manufacturer’s directions. Fresh stock solutions or samples stored at −70 °C were used for each experiment.

### 2.3. MIC Testing

MIC testing followed the method recommended by the Clinical and Laboratory Standards Institute [26]. Briefly, thawed isolates were sub-cultured twice on blood agar (BA) and incubated for 18–24 h in O_2_ at 35–37 °C. Mueller-Hinton Broth (MHB) (Difco Laboratories, Detroit, MI, USA), containing 2-fold drug concentration increments, was added to 96-well micro dilution trays. *E. coli*, *S. pseudintermedius*, *P. mirabilis*, and *E. faecalis* suspensions equal to a 0.5 McFarlane standard were diluted to achieve a final inoculum of 5 × 10^5^ cfu/mL and added to the microtiter trays, incubated for 18–24 h at 35–37 °C in O_2_, following which, the lowest drug concentration preventing visible bacterial growth was the MIC. The American Type Culture Collection (ATCC) strains *Enterococcus faecalis* 29212, *Escherichia coli* 25922, *Staphylococcus aureus* 29213, and *Pseudomonas aeruginosa* 27953 were tested with each MIC assay to ensure the assays were within acceptable performance ranges. MIC values for the 12 strains examined are shown in Table 1.

### 2.4. MPC Testing

Using a modified MPC protocol, 5 BA plates per isolate were inoculated for confluent growth, incubated for 18–24 h at 35–37 °C in O_2_, and following which, the complete contents of the inoculated plates were transferred to 100 mL of MHB and incubated (18–24 h at 35–37 °C in O_2_) [27,28]. Following incubation, cultures were estimated to have concentrations of ≥3 × 10^9^ cfu/mL by spectrophotometric readings (600 nm) ≥0.3 (Thermo Scientific GENESYS^TM^ 10S UV-Vis, Mississauga, ON, Canada) and by colony counts. Aliquots of 100 µL containing ≥10^9^ cfu were applied to antimicrobial agent containing BA plates over a range of drug concentrations: ampicillin 0.25–256 µg/mL; cephalexin 0.002–256 µg/mL; marbofloxacin 0.002–64 µg/mL; pradofloxacin 0.008–256 µg/mL; TMP/SMX 0.008/1.398–128/24–32 µg/mL. Drug plates were used within 1 week of preparation. Inoculated plates were incubated (as described) for a total of 48 h, with examination for growth at 24 and 48 h. The lowest drug concentration preventing growth (48 h) was the MPC. Each experiment included the 4 ATCC control strains summarized above. MPC values for the 12 strains examined are summarized in Table 1.

### 2.5. Kill Experiments

*S. pseudintermedius*, *E. coli*, *P. mirabilis*, and *E. faecalis* isolates were incubated for 18–24 h at 35–37 °C in O_2_ on BA, following which, an inoculum was transferred to MHB and incubated at 35–37 °C in O_2_ for 2 h. Following incubation, spectrophotometric readings of ≥0.3 verified cell densities ≥10^9^ cells/mL [27]. Adjusting of the inocula to achieve cell densities of 10^5^ cfu/mL was undertaken in MHB to which antimicrobial agent was added. Colony counts at time 0 for *E. coli*, *S. pseudintermedius*, *P. mirabilis*, and *E. faecalis* were as follows, respectively: ampicillin 1.03–9.8 × 10^5^ cfu/mL; cephalexin 1.27–9.67 × 10^5^ cfu/mL; marbofloxacin 1.07–8.93 × 10^5^ cfu/mL; pradofloxacin 1.00–8.87 × 10^5^ cfu/mL; TMP/SMX 1.07–9 × 10^5^ cfu/mL.

The drug concentrations used for the kill experiments were based on the measured MIC or MPC drug concentrations for each antimicrobial agent against each strain. The maximum serum (C_max_) and maximum urine (Urine_max_) drug concentrations were from published studies or reports for ampicillin [4], cephalexin [29], marbofloxacin [30], pradofloxacin (Bayer Animal Health (Elanco as of 2020, Monheim, Germany—data on file) and TMP/SMX [4] (Table 1). Killing (log_10_ reduction in viable cells and percentage of organism killed) was recorded at 5, 10, 15, 20, 25, 30, 60, 120, and 180 min following drug exposure by culturing aliquots on drug-free blood agar plates incubated for 18–24 h at 35–37 °C in O_2_. The log_10_ and percent kill reduction of viable cells were calculated and recorded. Killing was quantified by measuring the reduction in viable cell count from time 0 to the count at time 5 min after drug exposure and so on. Three separate aliquots were sampled at each time frame, and the results were averaged, as were the results for the 3 strains of each genus (*S. pseudintermedius*, *E. coli*, *P. mirabilis*, *E. faecalis*). As such, each datum point on the log_10_ reduction graphs represents the average of 9 individual measurements (i.e., measurements in triplicate and averaged for 3 strains).

### 2.6. Statistical Analysis

Statistical analysis (Statistical Analysis Software—SAS) of the data were performed using a repeated-measures ANCOVA for each drug data set, with fixed effects consisting of drug and drug-by-time interaction [22]. In each model, CFU count at time 0 was included as a covariate and a compound symmetric covariance structure was used. The transformed square root CFU counts were used to achieve a normal distribution. Bonferroni adjustments for multiple comparisons were made. Least square means were back-transformed and presented as log_10_ means. Values of *p* ≤0.05 (two-tailed) were considered significant for all analyses.

## 3. Results

The MIC and MPC values for the 12 bacterial strains tested in this study are shown in Table 1.

### 3.1. Escherichia coli

**Figure 1:**
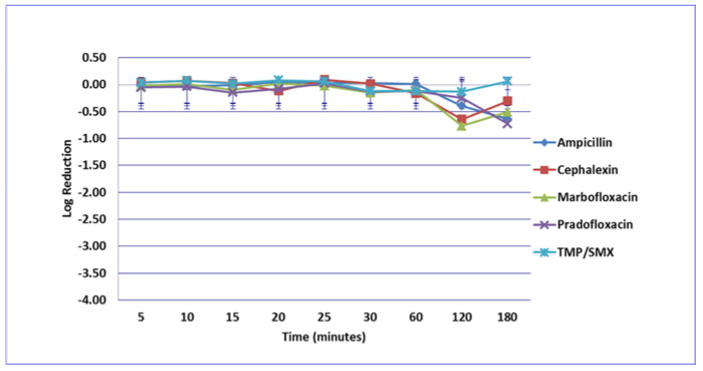
Log_10_ reduction in *E. coli* with bacterial cells exposed to ampicillin, cephalexin, marbofloxacin, pradofloxacin, and trimethoprim/sulfamethoxazole at the minimum inhibitory drug concentration. Data points present mean ± standard deviation.

**Figure 2:**
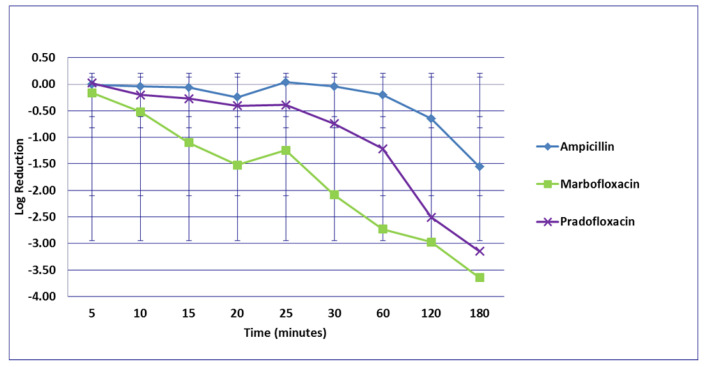
Log_10_ reduction in *E. coli* with bacterial cells exposed to ampicillin, marbofloxacin, and pradofloxacin at the mutant prevention drug concentration. Marbofloxacin versus ampicillin at 30 min *p* = 0.0289. Data points present mean ± standard deviation.

**Figure 3:**
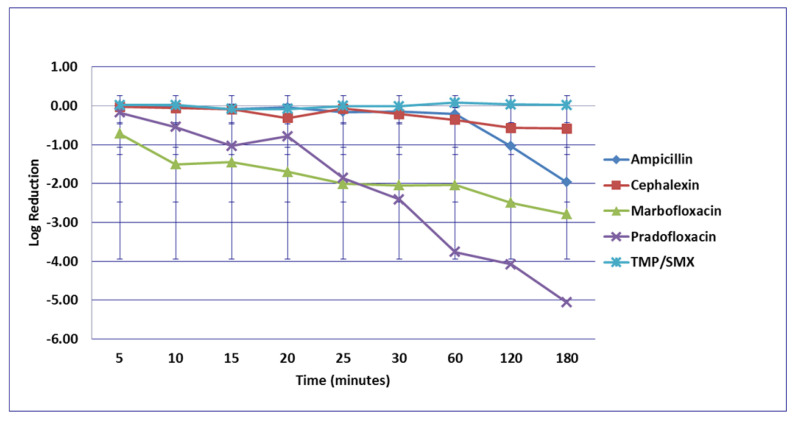
Log_10_ reduction in *E. coli* with cells exposed to ampicillin, cephalexin, marbofloxacin, pradofloxacin, and trimethoprim/sulfamethoxazole at the maximum serum drug concentration. *Pradofloxacin vs. TMP/SMX at 60, 120, and 180 min, *p* values = 0.0136, 0.0234, 0.0211. Marbofloxacin vs. TMP/SMX at 120, 180 min, *p* values = 0.0269, 0.0218. TMP/SMX vs. ampicillin at 180 min, *p* value = 0.0300. Data points present mean ± standard deviation.

**Figure 4:**
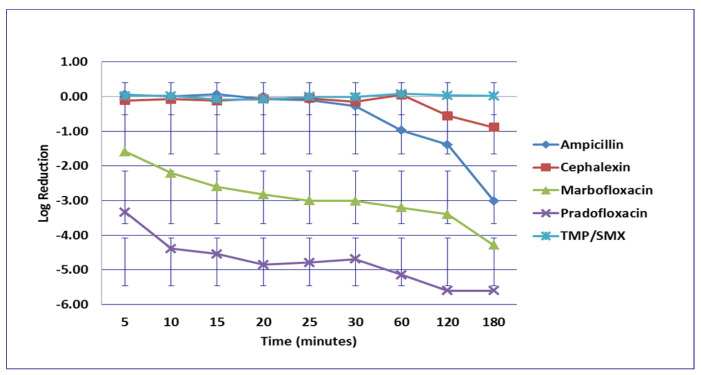
Log_10_ reduction in *E. coli* with bacterial cells exposed to ampicillin, cephalexin, marbofloxacin, pradofloxacin, and trimethoprim/sulfamethoxazole at the maximum urine drug concentration. Pradofloxacin vs. cephalexin at 5, 10, 15, 20, 25, 30, 60, and 120 min, *p* values <0.0001 for comparison at 5–60 min and *p* = 0.0104 at 120 min. Pradofloxacin vs. TMP/SMX at all time points *p* = <0.0001 for all comparisons. Pradofloxacin vs. ampicillin at 5, 10, 15, 20, 25, and 30 min, *p* values <0.0001 to *p* = 0.0002. Marbofloxacin vs. cephalexin at 5, 10, 15, 20, 25, 30, 60, 120, and 180 min, *p* values from <0.0001 to 0.0029. Marbofloxacin vs. TMP/SMX at 5, 10, 15, 20, 25, 30, and 60 min, *p* values from <0.0001 to 0.0002. Marbofloxacin vs. ampicillin at 5, 10, 15, 20, 25 and 30 min, *p* values from <0.0001 to 0.0002. TMP/SMX vs. ampicillin at 60, 120, and 180 min, *p* values from <0.0001 to 0.0028. Data points present mean ± standard deviation.

No significant differences in killing of the *E. coli* strains were seen among any of the drugs tested at the MIC drug concentration (Figure 1). At MPC drug concentration (Figure 2), marbofloxacin (2.09 log_10_, 96.7% kill) killed more bacteria than did ampicillin (0.04 log_10_, 8.3% kill) (*p* = 0.0289) following 30 min of drug exposure. Killing of *E. coli* by cephalexin and TMP/SMX at the MPC were not performed due to MPC values that exceeded clinically achievable drug concentrations. At the C_max_ drug concentration (Figure 3), more bacteria were killed by pradofloxacin (3.8 log_10_, 94% kill) than by TMP/SMX (growth in presence of drug) (*p* = 0.0136) following 60 min of drug exposure. At 120 and 180 min of drug exposure, more bacteria were killed by pradofloxacin (4.1 and 5.1 log_10_, 99.9–100% kill, *p* = 0.0234, *p* = 0.0211) and marbofloxacin (2.5 and 2.8 log_10_, 99.4 and 99.7% kill, *p* = 0.0269 and *p* = 0.0218) than by TMP/SMX (growth in presence of drug). More cells were killed by ampicillin (1.97 log_10_, 99.5% kill) than TMP/SMX (growth in presence of drug) following 180 min of drug exposure (*p* = 0.03). No other comparisons were significantly different although the log_10_ and percentage kill was higher for marbofloxacin and pradofloxacin over all time points when compared to ampicillin, cephalexin, and TMP/SMX. At the Urine_max_ drug concentrations (Figure 4), pradofloxacin killed more bacteria at all time points (3.3–5.6 log_10_, 99.4–100% kill, with *p* < 0.0001 for all comparisons) than did TMP/SMX (growth in presence of drug to 0.1 log_10_, growth to 17% kill). Pradofloxacin (3.3–5.6 log_10_, 99.4–100% kill, *p* = 0.0029–<0.0001) killed more cells than did cephalexin (0.1–0.9 log_10_, 23–54% kill) following 5–120 min of drug exposure, but not at 180 min of drug exposure. Pradofloxacin (4.5–4.8 log_10_, 99.9% kill) killed more cells than did ampicillin (growth to 40.4% kill) at the 15 and 30 min time points (*p* = 0.0002 and <0.0001). Marbofloxacin (1.6–4.3log_10_, 94–99.9% kill, with *p* < 0.0001 for all comparisons) killed more cells than did TMP/SMX (growth to 0.08 log_10_, 16% kill) at all time points and killed more cells than did cephalexin (growth in presence of drug to 0.55 log_10_, 53.4% kill) at the 5–120 min time points (*p* = 0.0006–<0.0001). Finally, cephalexin (0.9 log_10_, 82% kill, *p* = 0.0008) killed more cells than did TMP/SMX (growth in presence of drug) following 180 min of drug exposure.

### 3.2. Enterococcus faecalis

**Figure 5:**
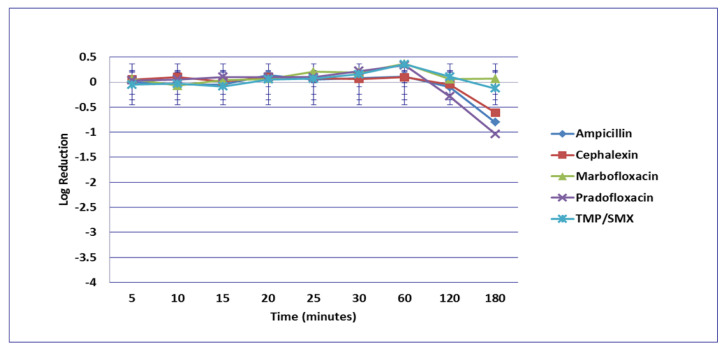
Log_10_ reduction in *E. faecalis* with bacterial cells exposed to ampicillin, cephalexin, marbofloxacin, pradofloxacin, and trimethoprim/sulfamethoxazole at the minimum inhibitory drug concentration. Data points present mean ± standard deviation.

**Figure 6:**
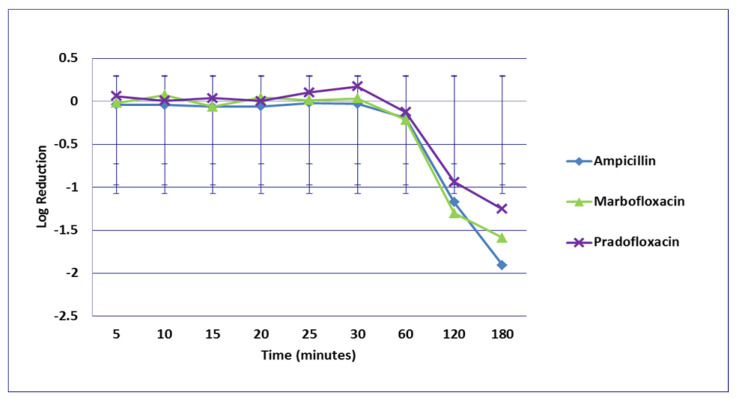
Log_10_ reduction in *E. faecalis* with bacterial cells exposed to ampicillin, marbofloxacin, and pradofloxacin at the mutant prevention drug concentration. Data points present mean ± standard deviation.

**Figure 7:**
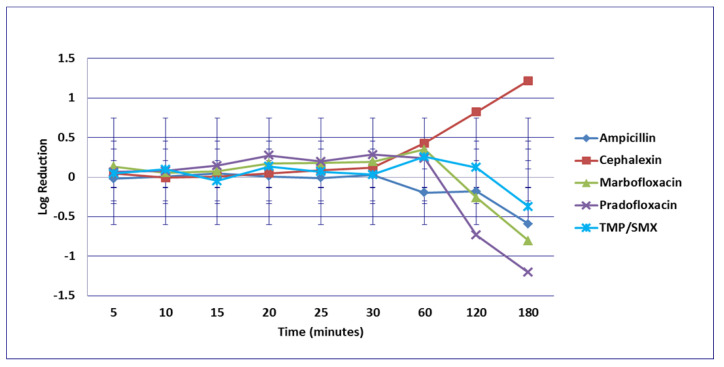
Log_10_ reduction in *E. faecalis* with bacterial cells exposed to ampicillin, cephalexin, marbofloxacin, pradofloxacin, and trimethoprim/sulfamethoxazole at the maximum serum drug concentration. Pradofloxacin vs. cephalexin at 120 and 180 min, *p* values <0.0001 for both comparisons. Marbofloxacin, TMP/SMX, and ampicillin versus cephalexin at 120 and 180 min, *p* value <0.0001 for all comparisons. Data points present mean ± standard deviation.

**Figure 8:**
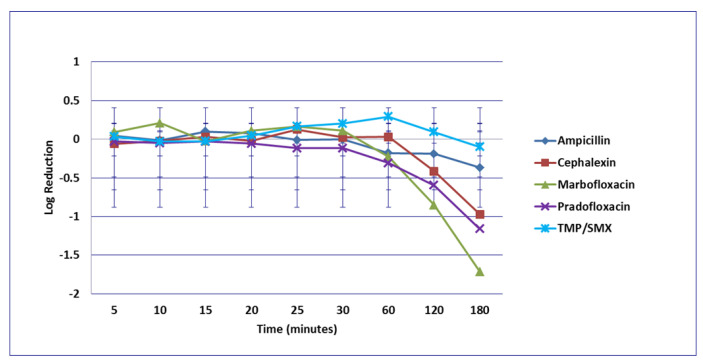
Log_10_ reduction in *E. faecalis* with bacterial cells exposed to ampicillin, cephalexin, marbofloxacin, pradofloxacin, and trimethoprim/sulfamethoxazole at the maximum urine drug concentration. Pradofloxacin vs. TMP/SMX at 60, 120, and 180 min, *p* values 0.0011, 0.0059, and 0.0004. Marbofloxacin vs. TMP/SMX at 60, 120, and 180 min, *p* values 0.0029, <0.0001, <0.0001. Data points present mean ± standard deviation.

For the *E. faecalis* strains investigated, significant differences were not seen among any drug comparisons at any time intervals at the MIC (Figure 5) and MPC (Figure 6) drug concentrations. Killing of *E. faecalis* by cephalexin and TMP/SMX at the MPC drug concentration was not done due to MPC values, exceeding clinically achievable drug concentrations. Following 120 min exposure at the C_max_ (Figure 7), more cells were killed by pradofloxacin (0.7 log_10_, 79% kill, *p* < 0.0001) than by TMP/SMX (growth in presence of drug) or by cephalexin (growth in presence of drug, *p* < 0.0001) and marbofloxacin (0.3 log_10_, 35% kill, *p* = 0.0007) killed more cells than did cephalexin (growth in presence of drug). Pradofloxacin (1.2 log_10_, 88% kill, *p* < 0.0001) killed more cells than cephalexin (growth in presence of drug), but not more cells than TMP/SMX (0.4 log_10_, 12% kill, *p* = 0.0654) after 180 min exposure. TMP/SMX (0.37 log_10_, 41.5% kill, *p* < 0.0001), and marbofloxacin (0.8 log_10_, 78.8% kill, *p* < 0.0001) killed more cells than did cephalexin (growth in presence of drug). At the Urine_max_ drug concentration (Figure 8), and following 60, 120, and 180 min of drug exposure, pradofloxacin (0.3, 0.6, 1.2log_10_, 50, 75, 92% kill, *p* = 0.0016, *p* = 0.0031, *p* = 0.0076), and marbofloxacin (0.2, 0.9, 1.7log_10_, 40, 86, 98% kill, *p* = 0.0031, *p* = 0.0001, *p* = 0.0001) killed more cells than did TMP/SMX (growth to 0.1 log_10_, 12% kill). Bacterial kill by ampicillin following 120 and 180 min exposure ranged from 33 to 56%.

### 3.3. Proteus mirabilis

**Figure 9:**
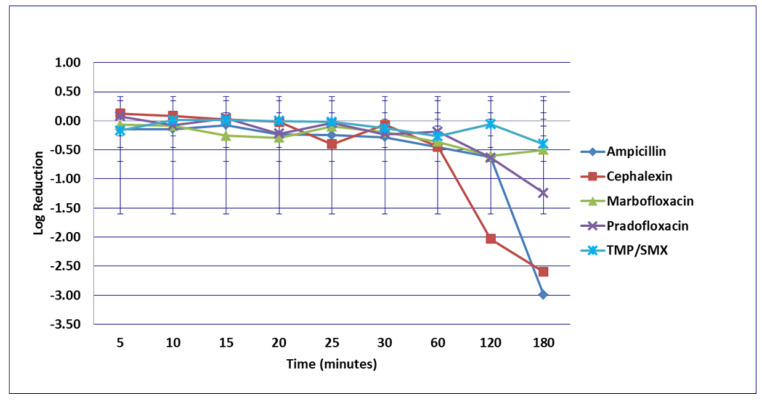
Log_10_ reduction in *P. mirabilis* with bacterial cells exposed to ampicillin, cephalexin, marbofloxacin, pradofloxacin, and trimethoprim/sulfamethoxazole at the minimum inhibitory drug concentration. Data points present mean ± standard deviation.

**Figure 10:**
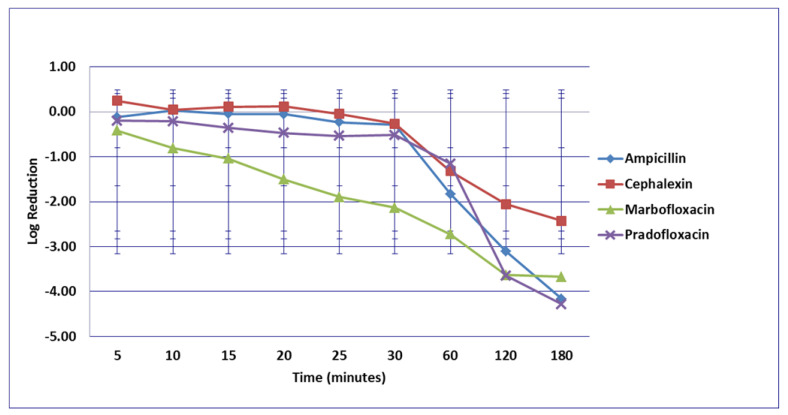
Log_10_ reduction in *P. mirabilis* with bacterial cells exposed to ampicillin, cephalexin, marbofloxacin, and pradofloxacin at the mutant prevention drug concentration. Marbofloxacin vs. ampicillin at 15, 20, 25, and 30 min, *p* values from 0.0006 to 0.0214. Data points present mean ± standard deviation.

**Figure 11:**
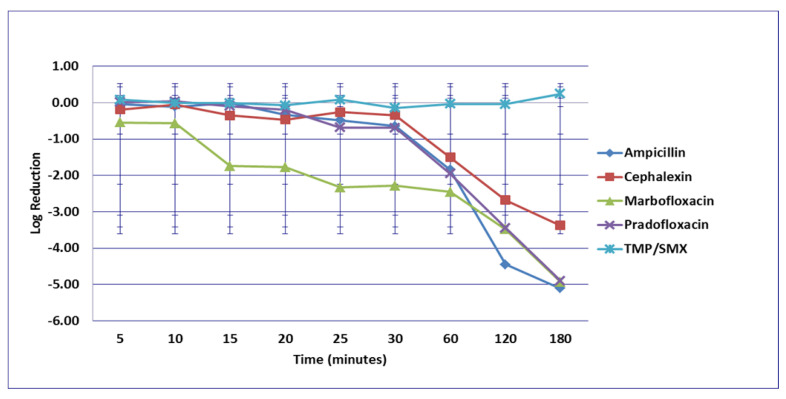
Log_10_ reduction in *P. mirabilis* with bacterial cells exposed to ampicillin, cephalexin, marbofloxacin, pradofloxacin, and trimethoprim/sulfamethoxazole at the maximum serum drug concentration. Pradofloxacin vs. marbofloxacin at 15 min, *p* = 0.0133. Pradofloxacin vs. TMP/SMX at 60, 120, and 180 min, *p* values 0.0058, 0.0009, <0.0001. Marbofloxacin vs. TMP/SMX at 15, 20, 25, 30, 60, 120, and 180 min, *p* values from <0.0001 to 0.0170. Cephalexin vs. marbofloxacin at 25 min, *p* = 0.0091. Cephalexin vs. TMP/SMX at 120 and 180 min, *p* = 0.0032 and <0.0001. TMP/SMX vs. ampicillin at 180 min, *p* <0.0001. Data points present mean ± standard deviation.

**Figure 12:**
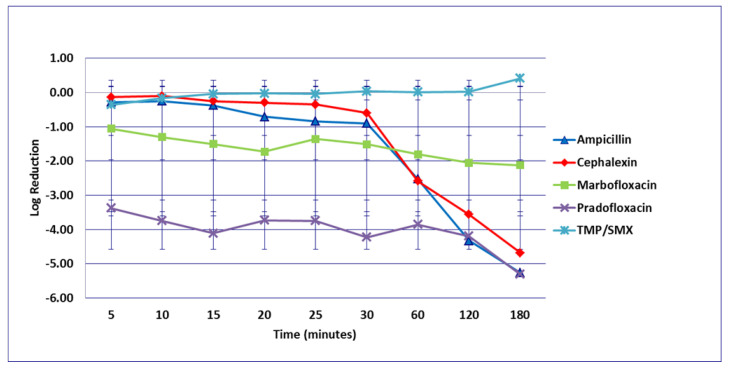
Log_10_ reduction in *P. mirabilis* with bacterial cells exposed to ampicillin, cephalexin, marbofloxacin, pradofloxacin, and trimethoprim/sulfamethoxazole at the maximum urine drug concentration. Pradofloxacin vs. cephalexin at 5, 10, and 15 min, *p* values from 0.0106 to 0.0483. Pradofloxacin vs. ampicillin at 5, 10, and 15 min, *p* values from <0.0001 to 0.0061. Pradofloxacin vs. TMP/SMX at 10, 15, 20, 25, 30, 60, 120, and 180 min, *p* values from <0.0001 to 0.0028. Marbofloxacin vs. TMP/SMX at 15, 20, 25, 30, 60, 120, and 180 min, *p* values from <0.0001 to 0.0434. TMP/SMX vs. ampicillin at 60, 120, and 180 min, *p* values <0.0001 for all comparisons. Data points present mean ± standard deviation.

For the *P. mirabilis* strains, no significant difference in kill was seen by any drug or at any time at the MIC drug concentration (Figure 9). At the MPC drug concentration (Figure 10), more cells were killed by marbofloxacin (0.42–2.73 log_10_, 64–99% kill) than by cephalexin (growth in presence of drug—1.32 log_10_, 83% kill) following 5 to 60 min of drug exposure (*p* = 0.0004–0.0045). Marbofloxacin (0.81–2.1 log_10_, 91.6–99.1% kill) killed more cells than did ampicillin (growth in presence of drug to 0.28 log_10_, 36.1% kill) following 10 to 30 min exposure (*p* = 0.0006–0.0214). At 120 and 180 min, 99.4 and 99.9% of cells were killed by marbofloxacin (3.63–3.67 log_10_) and pradofloxacin (3.6–4.3 log_10_) as compared to 80.3% by cephalexin (2.05–2.4 log_10_). Killing of *P. mirabilis* by TMP/SMX at the MPC drug concentration was not done due to MPC values exceeding the clinically achievable drug concentration. At the C_max_ drug concentrations (Figure 11), more cells were killed by marbofloxacin (1.8 log_10_, 99% kill) than by pradofloxacin (0.1 log_10_, 17% kill, *p* = 0.0495) or by TMP/SMX (0.01 log_10_, 6% kill, *p* = 0.0183) following 15 min exposure. Following 25 min exposure, more cells were killed by marbofloxacin (2.3 log_10_, 99.6% kill) than by cephalexin (0.3 log_10_, 68% kill, *p* = 0.0355) or by TMP/SMX (growth in presence of drug, *p* = 0.0011). Following 30 min of drug exposure, more cells were killed by marbofloxacin (2.3 log_10_, 99.4% kill) than by TMP/SMX (0.2 log_10_, 13% kill, *p* = 0.0414). Following 60, 120, and 180 min exposure, more cells were killed by ampicillin (1.8–5.1 log_10_, 97.1–99.9% kill) than by TMP/SMX (0.03 log_10_, 7% kill, to growth in presence of drug, *p* values from <0.0001–0.0102). At 60, 120, and 180 min of exposure, more cells were killed by pradofloxacin (2–5 log_10_, 96–99.9% kill) than by TMP/SMX (growth to 0.4 log_10_, growth to 7% kill, *p* = 0.0251–<0.0001) and the same was seen for marbofloxacin (3–5 log_10_, 99.6–100% kill) versus TMP/SMX (*p* = 0.0058–<0.0001). At 120 and 180 min, more cells were killed by cephalexin (3–3.4 log_10_, 99.2–99.9% kill) than by TMP/SMX (*p* = 0.0149–<0.0001). At the Urine_max_ drug concentrations (Figure 12), more cells were killed by pradofloxacin (3.4–5.3 log_10_, 99.4–100% kill) at 10 min of exposure and thereafter than by TMP/SMX (growth—0.4 log_10_, growth—53% kill, *p* = 0.0106–<0.0001). Pradofloxacin (3.7–4.1 log_10_, 99.6–99.9% kill) killed more cells than did cephalexin (0.13–0.25 log_10_, 21.3–46.4% kill) following 5, 10, and 15 min of exposure (*p* = 0.0483–0.0106). More cells were killed by marbofloxacin (1.5–2.1 log_10_, 96.8–99.3% kill) than by TMP/SMX (growth in presence of the drug to 0.04 log_10_, 10.4% kill) (*p* values from 0.0434–<0.0001) at 15 min of exposure and thereafter. More cells were killed by cephalexin (4–4.7 log_10_, 99.9% kill) than by TMP/SMX (*p* values from 0.0181–<0.0001) at 60, 120 and 180 min of exposure. At 5, 10, and 15 min exposure, pradofloxacin (3.7–4.1 log_10_, 99.4–99.9% kill) killed more cells than did ampicillin (0.28–0.38 log_10_, 7–54.8% kill) (*p* = 0.0004–<0.0001).

### 3.4. Staphylococcus pseudintermedius

**Figure 13:**
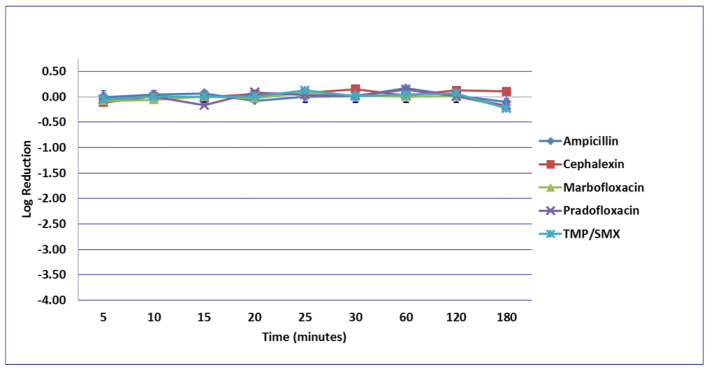
Log_10_ reduction in *S. pseudintermedius* with bacterial cells exposed to ampicillin, cephalexin, marbofloxacin, pradofloxacin, and trimethoprim/sulfamethoxazole at the minimum inhibitory drug concentration. Data points present mean ± standard deviation.

**Figure 14:**
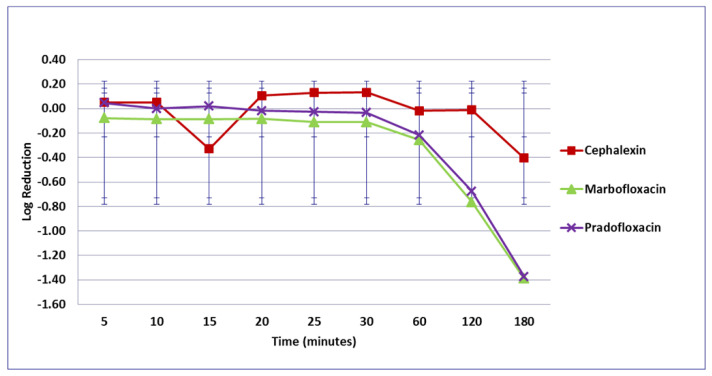
Log_10_ reduction in *S. pseudintermedius* with bacterial cells exposed to cephalexin, marbofloxacin, and pradofloxacin at the mutant prevention drug concentration. Data points present mean ± standard deviation.

**Figure 15:**
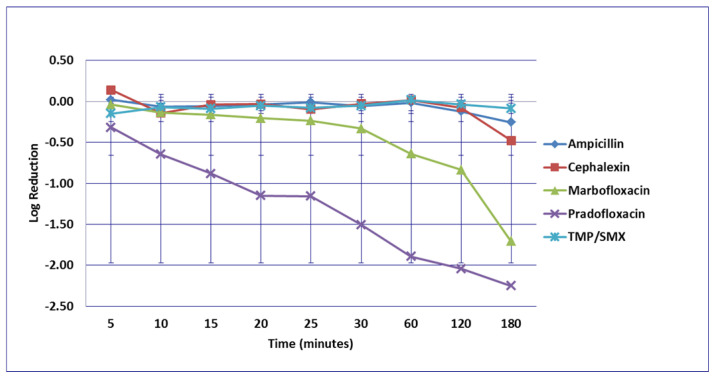
Log_10_ reduction in *S. pseudintermedius* with bacterial cells exposed to ampicillin, cephalexin, marbofloxacin, pradofloxacin, and trimethoprim/sulfamethoxazole at the maximum serum drug concentration. *Pradofloxacin vs. ampicillin at 25, 30, 60, and 120 min, *p* values from <0.0001 to 0.0045. Pradofloxacin vs. cephalexin at 20, 30, 60, and 120 min, *p* values from <0.0001 to 0.0394. Pradofloxacin vs. TMP/SMX at 30, 60, 120, and 180 min, *p* values from <0.0001 to 0.0017. Cephalexin vs. marbofloxacin at 60, 120, and 180 min, *p* values from 0.0006 to 0.0037. Marbofloxacin vs. TMP/SMX at 60, 120, and 180 min, *p* values from <0.0001 to 0.0033. Marbofloxacin vs. ampicillin at 60, 120, and 180 min, *p* values from <0.0001 to 0.0025. Data points present mean ± standard deviation.

**Figure 16:**
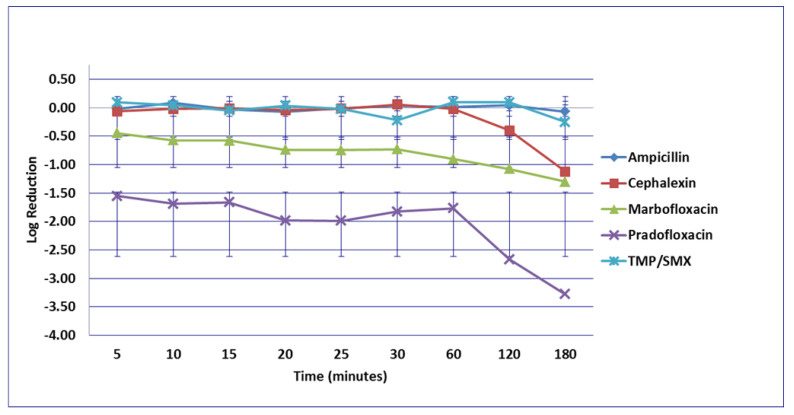
Log_10_ reduction in *S. pseudintermedius* with bacterial cells exposed to ampicillin, cephalexin, marbofloxacin, pradofloxacin, and trimethoprim/sulfamethoxazole at the maximum urine drug concentration. Pradofloxacin vs. cephalexin at 20 and 30 min, *p* values <0.0001 and 0.00474. Cephalexin vs. marbofloxacin at 20 min, *p* = 0.0009. Pradofloxacin vs. TMP/SMX at 60 and 120 min, *p* values 0.0489 and 0.0179. Pradofloxacin vs. ampicillin at 120 min, *p* = 0.0348. Data points present mean ± standard deviation.

For the *S. pseudintermedius* strains, no significant differences were seen among any comparisons or at any time point of the MIC (Figure 13), or the MPC drug concentration (Figure 14). Killing of *S. pseudintermedius* by TMP/SMX at the MPC was not done due to MPC values exceeding clinically achievable concentration. At the C_max_ (Figure 15) and after 20 min exposure, pradofloxacin (1.2 log_10_, 83% kill, *p* = 0.0394) killed more cells than did cephalexin 0.03 log_10_, 1% kill) and after 30 min exposure, pradofloxacin (1.5 log_10_, 93% kill) killed more cells than cephalexin (0.03 log_10_, 3% kill, *p* = 0.0006) or TMP/SMX (0.05 log_10_, 9% kill, *p* = 0.002). After 25–120 min exposure, pradofloxacin (1.2–2.3 log_10_, 85–97.6% kill) killed more cells than ampicillin (0.01–0.25 log_10_, 1.4–42.7% kill) (*p* values from 0.0045–<0.0001). After 60 min exposure, pradofloxacin (1.9 log_10_, 96% kill, *p* < 0.0001) killed more cells than cephalexin (growth in presence of drug) and more cells than TMP/SMX (growth in presence of drug, *p* < 0.0001); marbofloxacin (0.6 log_10_, 76% kill) killed more cells than cephalexin (*p* = 0.0029) and more cells than TMP/SMX (*p* = 0.0174). After 120 and 180 min exposure, marbofloxacin (0.83–1.7 log_10_, 81.7–97.6% kill) killed more cells than ampicillin (0.12–0.25 log_10_, 22–42.7%kill) (*p* = 0.0025 and <0.0001). After 120 min exposure pradofloxacin (2 log_10_, 97% kill) killed more cells than cephalexin (0.1 log_10_, 13% kill, *p* = 0.0002) and TMP/SMX (0.04 log_10_, *p* = 0.0001); marbofloxacin (0.8 log_10_, 82% kill) killed more cells than did cephalexin (*p* = 0.0087) and TMP/SMX (*p* = 0.0110). After 180 min exposure, pradofloxacin (2.3 log_10_, 98% kill) killed more cells than TMP/SMX (0.1 log_10_, 6% kill, *p* = 0.0001); marbofloxacin (1.7 log_10_, 98% kill) killed more cells than did TMP/SMX (*p* < 0.0001) and killed more cells than cephalexin (0.5 log_10_, 56% kill, *p* = 0.0017). At the Urine_max_ drug concentration (Figure 16), pradofloxacin (1.3 log_10_ and 1.8 log_10_, 90.1% and 93.6% kill) killed more cells than cephalexin (growth in presence of drug to 0.04 log_10_, 7.5% kill) following 20 and 30 min exposure (*p* = <0.0001, 0.0474), and more cells than TMP/SMX (growth in presence of drug) following 60 and 12 min exposure (*p* values 0.0489 and 0.0178). More cells were killed by pradofloxacin (2.7 log_10_, 97.3% kill) than by ampicillin (growth in presence of drug), following 120 min exposure (*p* = 0.0348).

## 4. Discussion

Urinary tract infections remain a common problem in dogs (less in cats) for which owners bring their pets to veterinarians and a urine sample for culture and susceptibility testing may be collected and submitted to a diagnostic laboratory. However, due to costs associated with laboratory investigations, owners may decline testing [31] and antimicrobial agents prescribed empirically. Without the benefit of the eventual results from culture confirming the infecting pathogen(s), the empiric drug choice may not be appropriate (i.e., against a susceptible organism). In both human [32] and veterinary medicine [33], the vast majority of antimicrobial agents are prescribed empirically. Local susceptibility data in the form of annual antibiograms provide relevant information that can aid empiric antimicrobial choices as can susceptibility data from surveillance studies; however, animal specific pathogens and susceptibility/resistance data is optimal.

MPC values of the analyzed pathogens were determined against quinolone and non-quinolone antibacterial agents. Others [34,35] have suggested MPC measurements do not apply to non-quinolone antibacterial agents as the main mechanisms of resistance is not related to point mutations. To this point, the terminology of “mutant prevention” [17,19] may be inadequate and we have previously commented “resistance” prevention may be a more appropriate terminology when considering various classes of antimicrobial agents. Regardless, just as a MIC is the drug concentration blocking the least susceptible cell present in a bacterial density of 10^5^ cfu/mL, MPC or (RPC) is the drug concentration blocking the growth of the least susceptible cell in a higher bacterial density (~10^9^ cfu or higher) regardless of mechanism(s) of reduced susceptibility/resistance. Such high bacterial burdens are reported in a number of infections including pneumonia [36], respiratory tract infections [37], meningitis [37,38], and urinary tract infections [39,40].

We investigated in vitro killing or inhibition of key canine urinary pathogens by ampicillin, cephalexin, marbofloxacin, pradofloxacin, and TMP/SMX within the first 3 h after drug exposure utilizing clinically relevant drug concentrations. Significant differences were seen between agents depending on the time of sampling and drug concentrations tested. The two quinolones and TMP/SMX were chosen for these measurements as these agents have been previously recognized as clinically useful agents for short course therapy in humans. Amoxicillin (or ampicillin) was chosen for inclusion in this study despite some limitations and concerns expressed in peer reviewed papers [14,41,42]. First, amoxicillin is not a candidate agent for short course therapy and in humans, aminopenicillins and first generation cephalosporins have lower efficacy [43,44], and are no longer recommended as first line therapy due to higher resistance rates and higher recurrences; however, these drugs can be used based on susceptibility results. The 2011 IDSA Clinical Practice Guidelines for acute uncomplicated cystitis specifically highlighted to avoid ampicillin or amoxicillin alone due to lower efficacy and that close follow up would be required [11]. How observations of inferior efficacy in human relates to use in companion animals requires further investigation and the absence of randomized control trials leaves this as an unanswered question. Cephalexin, a first generation cephalosporin, currently retains higher susceptibility than ampicillin/amoxicillin and was included for that reason despite it not being a candidate drug for short course therapy based on human data.

Second, *E. coli* resistance to amoxicillin/ampicillin have been increasing and in some studies exceed 40% from humans [45] and have higher resistance rates with inpatient isolates [46]. *E. coli* resistance to ampicillin/amoxicillin is variable in veterinary medicine and this organism remains amongst the most common urinary tract pathogen in male and female dogs [7,47,48,49]. Amoxicillin is a recommended first line option in the most recent ISCID guidelines for bacterial urinary tract infections in dogs and cat [1] despite reports of increasing or high *E. coli* resistance. The recommended duration of therapy is 3–5 days.

Chang et al. (2015) reported amoxicillin resistant *E. coli* rates of 27–57% from two centers in Taiwan and MIC_90_ values were >1024 µg/mL, well above peak or sustainable urine concentration. Similarly, in Iran, amoxicillin resistant *E. coli* represented 60–81% infections in dogs with a UTI [50]. Interestingly, *E. coli* strains isolated from healthy dogs were 38–73% resistant to amoxicillin. In the USA, 37–54% and 32–45% ampicillin resistance for *E. coli* was reported [48,51]. In the above studies, resistance to a first generation cephalosporin ranged from 0 to 34%, 10 to 44% for TMP/SMX, and 0 to 30% for fluoroquinolones. Guidelines for empiric therapy suggest that antibiotic choices should be based on local susceptibility data and the resistance to a drug should be ≤20%.

The types of drugs used for urinary tract infection in companion animals varies. Indeed, a survey of ~3000 practitioners in 25 European countries reported penicillins accounted for 55% of use in dogs for urogenital infection followed by fluoroquinolones (26%), first and second generation cephalosporins (9%) and potentiated sulphonamides (6%) [52]. In contrast, a report on antibiotic use patterns in dogs at a veterinary teaching hospital indicated that most frequently prescribed antibiotics for therapeutic reasons were amoxicillin-clavulanic acid (24.7%), cefazolin/cephalexin (18.3%), enrofloxacin (16.2%), ampicillin/amoxicillin (14.9%), and doxycycline (11.2%) [33]. Of antibiotics used without documented evidence of infections, doxycycline (58.5%) was number one followed by cefazolin/cephalexin (37.8%), ampicillin/amoxicillin (37.3%), enrofloxacin (34%), and amoxicillin–clavulanate (30.8%).

The bactericidal properties of ampicillin [53], cephalexin [54,55], marbofloxacin [30], and pradofloxacin [22,56] have been previously reported, as has the bacteriostatic properties of TMP/SMX [55]. For example, marbofloxacin at twice the MIC for *S. pseudintermedius* showed a 2 log_10_ reduction in viable cells after 2 h of exposure, which increased to a >3 log_10_ reduction 6 h of drug exposure [30]. According to a previous study, where increasing concentration of pradofloxacin in kill assays resulted in a faster rate of kill and for strains of *E. coli*, *S. pseudintermedius* and *P. multocida*, bacterial activity was seen with pradofloxacin at drug concentrations of ≤0.25 µg/mL [57]. In our study, marbofloxacin and pradofloxacin exhibited rapid bactericidal activity with killing of *E. coli* occurring within minutes of exposure at MPC, C_max_, and urine drug concentrations. This observation is consistent with a concentration dependent drugs; the longer time to a bactericidal effect for ampicillin and cephalexin is consistent with a time-dependent agent. TMP/SMX is also a time-dependent drug but bacteriostatic.

Considerable debate has occurred regarding the use and potential differences between either bactericidal or bacteriostatic antimicrobial agents [24,58], and a recent systemic literature review [25] concluded no clinical intrinsic superiority of bactericidal agents compared to bacteriostatic agents. Regardless, these designations continue to be used based on log_10_ reduction in viable organisms in the presence of different drugs. In the present study, ampicillin, cephalexin, marbofloxacin, and pradofloxacin are all considered bactericidal agents and TMP/SMX is considered bacteriostatic. Under the experimental conditions in this report, TMP/SMX displayed bacteriostatic properties; that is, having log_10_ reductions in viable cells of <2 log_10_ based on the classic definition, whereas the other four agents showed bactericidal properties having a ≥3 log_10_ reduction in viable cells, depending on the drug concentrations tested [20,23]. Despite the differentiations between bactericidal and bacteriostatic agents, fluoroquinolones and TMP/SMX are recognized agents for short course therapy in uncomplicated cases and supported by clinical outcome data in humans.

The bacterial kill in our study was fastest with the tested fluoroquinolones (MPC, C_max_ and Urine_max_) with rapid reduction in viable cells within minutes of drug exposure. In some instances, and for some drug comparisons, significant differences maintained over the tested time intervals, while for others, such differences were lost. This likely relates to concentration and time-dependent agents being compared in the kill assays. Studies like this are unable to simulate drug elimination over time or the contributions of natural defenses and immunological responses to the recovery from infection in patients. Rapid killing within minutes versus hours may contribute to the clinical response or cure seen for some agents during short course therapy.

Limited data exist on the duration of therapy for UTIs in dogs with various antimicrobial agents and older recommendations were for therapy duration of ≥7 days; longer than those for humans with uncomplicated cystitis [8]. However, this was changed in the updated UTI guidelines [1]. An older study in humans compared single dose enoxacin therapy (600 mg SID) to 3-day treatment (200 mg BID) with the same drug [59]. Of 154 patients enrolled, 73 had positive pre-treatment urine cultures (≥10^5^ cfu/mL), of which 33 received single dose therapy and 40 received the 3-day course. *E. coli* was the most common pathogen detected. A cure was seen in 25/33 (76%) of single dose patients (negative urine culture) and 32/36 (89%) of 3-day treatment patients at 7–10 days and 18/33 and 27/36 at 4–6 weeks post-treatment, and the differences were not significant. This study suggests that short course therapy especially with concentration dependent antimicrobials is unlikely to compromise therapy outcome in uncomplicated cystitis.

The clinical and bacteriological success of pradofloxacin at 3 mg/kg orally, once daily for 3 days of therapy for uncomplicated urinary tract infections in dogs was evaluated [60]. In that study, 35 client owned dogs with symptoms of UTI were screened and 51% (n = 19) had a positive urine culture and were included in the study and the remainder excluded from inclusion. *E. coli* was recovered from 52% of dogs, followed by *S. pseudintermedius* (20%), *Proteus* spp. (15%), and *Klebsiella* spp. (10%). *Enterobacter* spp., *Enterococcus* spp. and beta-haemolytic *Streptococci* were recovered in approximately 3% each of animals. Clinical and bacteriological cure rates were not statistically different between treatments, for 3 days versus 7 days with pradofloxacin.

What is the antimicrobial treatment duration in patients with mild to moderate uncomplicated infection? Llewelyn and colleagues argued if the “antibiotic course has had its day” and “complete the course” is actually a barrier to antibiotic conservation [61]. Duration of therapy (based on randomized controlled trials) has not been uniformly established for all antimicrobials and all clinical indications. This is particularly true in veterinary, especially small animal, medicine where drug use is often extrapolated from human data. In humans, shorter-course antibiotic therapy has been evaluated with standard therapy ranging from 7 to 15 days and short course therapy ranging from 3 to 8 days [62,63,64]. In many instances, the length of therapy was reduced by 2–10 days suggesting that extended durations of therapy are unnecessary at least for some clinical indications. Short course therapy for uncomplicated UTI is standard in human medicine and even for serious infections, such as pyelonephritis, as 7 days of ciprofloxacin therapy [65], or once daily levofloxacin for 5 days, were not inferior to ciprofloxacin, given twice daily for 10 days for treatment of complicated UTI or acute pyelonephritis [66].

This study is unique in that it utilizes antimicrobial drug concentrations that are clinically relevant versus studies where drug concentrations are multiples (i.e., 2×, 4×, etc.) of the MIC drug concentration. Measuring drug concentrations that are not therapeutically relevant may be misleading. This study also has some limitations worthy of consideration. First, this was an in vitro investigation comparing bacterial killing by antimicrobial agents under controlled environments. Such studies are unable to incorporate drug concentration fluctuations that occur clinically nor consider the impact of immune responses, which remain largely under investigated [67], but progress in understanding bladder immunity is intriguing [68]. Second, the study was conducted over a defined time frame (5–180 min) and with a predetermined density of bacteria. Bacterial densities may be higher or lower (hence, MIC and MPC measurements) during infection and clinical drug therapy duration is longer than tested here. Despite the mentioned limitations, such studies are important in defining reference points and indicating differences among drugs under controlled conditions and, consequently, serve to support clinical trial planning, investigations, and observations.

The data from this study show more rapid reduction in viable bacterial cells with marbofloxacin and pradofloxacin against UTI pathogens at clinically relevant urinary tract drug concentrations. As important, reduction in bacterial counts were seen with all drugs by 180 min. The demonstrated killing by cephalexin appears consistent with a time-dependent drug. More clinical drug comparisons focusing on length of therapy and clinical cure and supported by in vitro investigations, as reported here, are essential for reducing unnecessarily long therapy regimens for uncomplicated infections, and are consistent with One Health and antimicrobial stewardship goals [69,70].

## Figures and Tables

**Table 1:** Comparative MIC, MPC, and drug concentration values.

Drug	Isolates						C_max_	Urine_max_
	#1		#2		#3			
	MIC	MPC	MIC	MPC	MIC	MPC		
*E. coli*								
Ampicillin	8	32	8	32	4	32	87	309
Cephalexin	8	≥128	8	≥128	8	≥128	20.3	225
Marbofloxacin	0.031	0.5	0.016	0.5	0.031	0.5	2.1	49.73
Pradofloxacin	0.031	0.125	0.25	0.5	0.016	0.125	1.4	237.9
TMP/SMX	0.25/4.75	≥8/152	0.125/2.38	≥8/152	0.031/0.595	≥8/152	1.55/29.45	26/79
*E. faecalis*								
Ampicillin	1	4	NT	NT	1	4	87	309
Cephalexin	128	≥256	128	≥256	128	≥256	20.3	225
Marbofloxacin	2	8	2	8	2	8	2.1	49.73
Pradofloxacin	0.25	2	0.25	1	0.5	2	1.4	237.9
TMP/SMX	0.25/4.75	≥8/152	0.125/2.38	≥4/76	0.125/2.38	≥4/76	1.55/29.45	26/79
*P. mirabilis*								
Ampicillin	1	64	1	64	2	64	87	309
Cephalexin	16	64	16	128	16	64	20.3	225
Marbofloxacin	0.031	1	0.031	1	0.063	1	2.1	49.73
Pradofloxacin	0.063	1	0.25	1	0.125	1	1.4	237.9
TMP/SMX	0.125/2.38	≥8/152	32/608	≥8/152	128/2432	≥8/152	1.55/29.45	26/79
*S. pseudintermedius*								
Ampicillin	0.125	NT	1	NT	2	NT	87	309
Cephalexin	2	64	0.5	16	0.5	16	20.3	225
Marbofloxacin	0.25	0.5	0.063	0.5	0.25	0.5	2.1	49.73
Pradofloxacin	0.031	0.125	0.016	0.063	0.031	0.125	1.4	237.9
TMP/SMX	0.5/9.5	≥8/152	0.5/9.5	≥8/152	1/19	≥8/152	1.55/29.45	26/79

MIC = minimum inhibitory concentration; MPC = mutant prevention concentration; C_max_ = maximum serum drug concentration, Urine_max_ = maximum urine drug concentration; µg/mL = microgram per milliliter; NT = not tested.

## Data Availability

The data that support the findings of this study are available from the corresponding author upon reasonable request.
